# Negative pressure wound therapy reduces the incidence of postoperative wound dehiscence and surgical site infections after total knee arthroplasty in patients with obesity

**DOI:** 10.1097/MD.0000000000029641

**Published:** 2022-07-08

**Authors:** Qi-Chun Song, Dong Li, Yan Zhao, Guang-Yang Zhang, Dong-Long Shang, Li-Hong Fan, Xiao-Qian Dang

**Affiliations:** a The Second Affiliated Hospital of Xi’an Jiaotong University, Xi’an, People’s Republic of China.

**Keywords:** negative pressure wound treatment, obesity, surgical site infection, total knee replacement, wound dehiscence

## Abstract

Obesity is a risk factor for total knee arthroplasty (TKA). Wound dehiscence and surgical site infections (SSIs) are the main complications of TKA in patients with obesity. They can profoundly affect patients because they often require readmission, additional surgical interventions, lengthy intravenous antibiotic administration, and delayed rehabilitation. Negative pressure wound therapy (NPWT) exposes the wound site to negative pressure, resulting in the improvement of blood supply, removal of excess fluid, and stimulation of cellular proliferation of granulation tissue. This study aims to assess the incidence of wound dehiscence and SSIs in patients with obesity undergoing TKA after the routine use of NPWT. This sduty enrolled adult patients with obesity who underwent TKA within 8 years. A total of 360 adult patients with obesity (NPWT: 150, non-NPWT: 210) underwent TKA, and the baseline characteristics were similar between the 2 groups. Compared with the non-NPWT group, the NPWT group had a 50% lower incidence of wound dehiscence (3.33% vs 9.52%; *P* < .05) and a significantly lower incidence of SSIs (11.33% vs 25.24%; *P* < .05), including prosthetic joint infection (4.0% vs 10.0%; *P* < .05) and superficial wound infection (7.33% vs 15.24%; *P* < .05). In addition, the NPWT group had a lower need to return to the operating room for new interventions for any reason (2.67% vs 9.05%; *P* = .0107) than the non-NPWT group. Conventional incision NPWT can significantly reduce the incidence of wound dehiscence and SSIs in patients with obesity after TKA.

## 1. Introduction

Obesity is a risk factor for osteoarthritis. The prevalence of total knee arthroplasty (TKA) in individuals with obesity has increased in recent years and is expected to grow exponentially in the coming decades.^[1,2]^ Despite the use of prophylactic antibiotics, wound dehiscence and surgical site infections (SSIs) remain serious problems in patients with obesity.^[3,4]^ SSIs interfere with the normal cellular mechanism of wound healing and potential tissue denaturation, leading to tissue rupture and wound dehiscence. Invasive tissue mobilization is used to facilitate the closure of wounds without proper care to protect the blood supply in the skin; however, this technique can lead to dehiscence and infection because denatured tissue is a target site of bacterial infection.^[5]^ This phenomenon is especially true for the wounds of patient with obesity, where space containing metal hardware may be surrounded by extravascular tissue. Wound dehiscence and SSIs can profoundly affect patients because they often require readmission, additional surgical interventions, and lengthy intravenous antibiotic administration and delayed rehabilitation.

Negative pressure wound therapy (NPWT) was proposed in the early 1990s for the treatment of chronic wounds.^[6]^ The effectiveness of NPWT in chronic wounds, pressure ulcers, and diabetic foot ulcers is well recognized. In the past 2 decades, the role of NPWT has been extended to promote wound healing and reduce the incidence of complications after elective surgery.^[7,8]^ At present, NPWT have been widely used in other surgical disciplines and have achieved good clinical efficacy.^[9,10]^ Closed-incision negative pressure therapy is usually applied after total hip and knee arthroplasty when postoperative infection and surgical site complications occur.^[11]^ Therefore, the present study aimed to compare the incidence of wound dehiscence and SSIs between patients with obesity receiving TKA and using portable NPWT immediately after surgery and those using conventional dressings (control group). Our hypothesis is that NPWT reduces the incidence of wound dehiscence and SSIs in patients with obesity.

## 2. Methods

### 2.1. Patients

The hospitalization records of adult patients with obesity who underwent TKA in The Second Affiliated Hospital of Xi’an Jiaotong University from January 2013 to January 2021 were retrospectively analyzed. The inclusion criteria were patients with obesity over 18 years old with knee osteoarthritis who need to undergo selective unilateral arthroplasty. According to the World Health Organization classification, obesity is defined as the body mass index (BMI) >30 kg/m^2^. Exclusion criteria were patients who underwent knee, femur, or tibia surgery on the side of the replacement, patients with previous osteomyelitis on the same side of the femur or tibia as the knee and patients with severe deformity or previous severe ligament instability requiring revision implants on the tibia or femur. In addition, patients who could not perform the weekly postoperative evaluation in person according to the established schedule were excluded. The demographic, clinical manifestations, comorbidity including diabetes, hypertension and tobacco use, radiologic data and surgical wound healing complications of each patient were reviewed. The study was approved by the Medical and Biological Ethics Committee of Xi’an Jiaotong University Health Science Centre, and all participants signed the informed consent to the clinical study.

### 2.2. Treatment and control group

In 2017, the chief physician (X-QD) utilized the incision NPWT device routinely after TKA for patients with obesity. Before 2017, NPWT was not used. The control group (non-NPWT group) included patients who received only primary wound closure and standard dressing coverage after surgery and drainage (January 2013 to January 2017). The treatment group (NPWT group) included patients who used NPWT after primary wound closure (January 2017 to January 2021).

### 2.3. Operation information and negative pressure device placement

**Figure 1.**
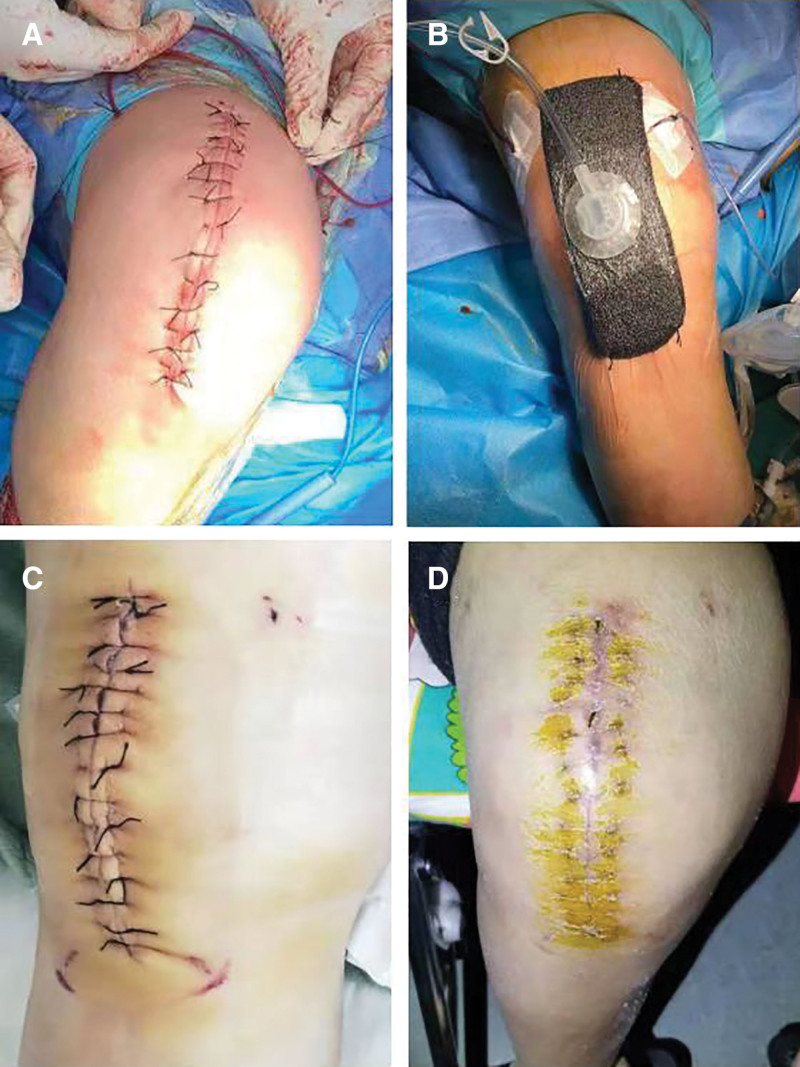
Wound closure and negative pressure device for TKA in obese patients. (A) Wound closure after TKA; (B) covering the NPWT immediately; (C) NPWT was removed 3 d later; (D) wounds were reviewed 1 mo later. TKA = total knee arthroplasty, NPWT = negative pressure wound therapy.

All cases used the same technology for the same type of intervention. The operations were performed by pneumatic tourniquet through anterior knee incision and quadrilateral and medial parapatellar approach. The surgery was always to remove the posterior cruciate ligament. All cases were treated with bone cement without antibiotics. In all operations, absorbable 2-0 Vicryl sutures were used to close the wound in the fascia and subcutaneous layer, and a 4-0 nylon suture was used to close the skin. All cases were treated with subfascial drainage. In the NPWT group, negative pressure devices (PICO^®^, Smith Nephew, London, United Kingdom) were used in addition to wound closure. The system consists of a small portable negative pressure pump and a dressing with a fixed film. The dressing can be adapted to the size of the incision and placed in the incision area. The fixed film was applied to the incision and around the wound to seal the wound. Continuous appropriate negative pressure of 75 to 85 mm Hg was applied to create a uniform negative pressure throughout the incision and draw excess wound fluid from the wound to the dressing. The drainage tube was removed on the second day after the operation regardless of the volume of drainage. In the NPWT group, the negative pressure device was maintained for 3 days (Fig. 1).

### 2.4. Standard preoperative and postoperative systemic prophylactic antibiotic regimens

All patients received standard systemic antibiotic prophylaxis within 1 hour before the incision, including weight based intravenous cefotiam, followed by intravenous cefoxitone sodium every 8 hours for 1 day. If the patient was allergic to penicillin, clindamycin was administered instead based on the body weight. Before skin sealing, pulse lavage was performed with 3 L normal saline.

### 2.5. Postoperative management and rehabilitation

The dressing was changed every 3 to 5 days after drainage tube removal in the non-NPWT group and after the negative pressure device was removed in the NPWT group, unless wound dressing was permeated and replaced immediately. The sutures of the patients in both groups were removed from the 14th day to the 21st day after the operation according to the date of outpatient return. From 1 day after the operation, the patients in the 2 groups gradually carried out range of motion exercises and weight-bearing because the application of the NPWT device will not hinder the patient’s functional exercise. Anti-coagulants were used to prevent venous thromboembolism for 35 days. Routine analgesics and opioids were used for pain control, if necessary.

### 2.6. Clinical function assessment

The American Knee Society Knee Score (AKSS) was used to evaluate the functional recovery at 3 months, 6 months, and 1 year after the operation. The score was divided into clinical and functional scores, including 50 points for pain, 25 points for a range of motion, 25 points for stability, 50 points for flexion and extension or lateral deformity, 50 points for walking, 50 points for going up and down stairs, and 200 points for crutches.

### 2.7. Clinical complications collection and evaluation

All surgical wound healing complications were evaluated weekly in the first 6 weeks. The presence or absence of all complications related to the wound were performed by 3 evaluators. The complications were suspected based on clinical history, physical examination, laboratory analysis and/or bacterial culture, radiologic analyses, and so on. The following complications related to the surgical wound healing were evaluated: dehiscence of the surgical wound (length exceeds 1 stitch pitch); SSIs, presence of a hematoma around the wound or operated knee; persistent drainage (considered as drainage for more than 7 days); presence of blisters around the surgical wound; the incidence of lower extremity deep vein thrombosis was also recorded; reintervention was considered as the need to take the patient to the operating room for any reason related to the arthroplasty.

SSIs include superficial and deep wound infection according to the Centers for Disease Control and Prevention Guideline.^[12]^ Deep wound infection is also considered prosthetic joint infection (PJI). The definition of PJI (deep SSI) and the difference between superficial and deep SSI developed by the 2nd International Consensus Meeting in Philly were considered to make a diagnosis.^[13]^ All patients in the study had standard laboratory tests on admission, including erythrocyte sedimentation rate, peripheral white blood cell count, C-reactive protein, complete urinalysis, microbiology, and blood culture. Erythrocyte sedimentation rate was determined by Wechsler method, and those over 15 mm/h were regarded as abnormal. C-reactive protein concentrations >5 mg/dL were considered abnormal.

### 2.8. Cost analysis

The total hospitalization expenses including operation expenses, material expenses, and hospitalization expenses were counted. The cost of complications or return to surgery during the review period was also included.

### 2.9. Statistical analysis

Baseline characteristics, surgical data, and SSI rates of each group were compared using the Mann–Whitney *U* test and *t* test continuous variables. Parameter data were expressed as mean ± standard deviation and compared using 2 independent samples *t* test. Non-parametric data were expressed as median [quartile range] and compared using the Mann–Whitney *U* test. Nominal data were compared using the χ^2^ test. Statistical significance was considered at *P* < .05.

## 3. Results

### 3.1. Baseline characteristics

Statistical analysis of 360 patients with obesity (NPWT: 150 cases, non-NPWT: 210 cases) undergoing TKA. The mean age was 63.23 ± 12.57 years (NPWT: 62.83 ± 13.14 years and non-NPWT: 63.52 ± 11.95 years), including 115 males and 245 females. In total, 68 cases (18.89%) of smoking, 176 cases (48.89%) of hypertension, and 69 cases (19.17%) of diabetes were recorded. The mean BMI was of patients was 33.46 ± 6.88. The 2 groups showed no significant differences. The baseline characteristics are summarized in Table 1.

**Table 1:** Comparison of demographic and comorbidity data between NPWT group and non-NPWT group.

Variables	NPWT group (n = 150)	Non-NPWT group (n = 210)	*P*
Mean age (y)	62.83 ± 13.14	63.52 ± 11.95	.5989
Male (%)	49 (32.67)	66 (31.43)	.8038
BMI (kg/m²)	34.15 ± 7.18	32.97 ± 6.64	.1090
Smoker (%)	27 (18.00)	41 (19.52)	.7157
Hypertension (%)	79 (52.67)	97 (46.19)	.2256
Diabetes (%)	32 (21.33)	37 (17.62)	.3774

Data presented as average (standard deviation) or number of subjects (percent of group).

BMI = body mass index, NPWT = negative pressure wound therapy.

### 3.2. Postoperative complications

The complications observed in each group are summarized in Table 2. The probability of superficial SSIs was 11.94%, with 7.33% in the NPWT group and 15.24% in the non-NPWT group (*P* = .0226). The probability of PJI was 7.5%, with 4.0% in the NPWT group and 10.0% in the non-NPWT group (*P* = .0331). The probability of wound dehiscence was 6.94%, with 3.33% in NPWT group and 9.52% in the non-NPWT group (*P* = .0227). In addition, the NPWT group had a lower need to return to the operating room for new interventions for any reason (2.67% vs 9.05%; *P* = .0107), but a higher incidence of blisters (22.67% vs 10.48%; *P* = .0017) than the non-NPWT group. No significant difference in the incidence of hematoma, continuous drainage, and deep venous thrombosis were found between the 2 groups.

**Table 2:** Comparison of postoperative complication rates between NPWT group and non-NPWT group.

Variables	NPWT group (n = 150)	Non-NPWT group (n = 210)	*P*
Dehiscence (%)	5 (3.33)	20 (9.52)	**.0227**
Superficial wound infect (%)	11 (7.33)	32 (15.24)	**.0226**
Prosthetic joint infect (%)	6 (4.00)	21 (10.00)	**.0331**
Persistent drainage (%)	4 (2.67)	13 (6.19)	.1202
Hyperemia (%)	29 (19.33)	63 (30.00)	**.0222**
Blister (%)	34 (22.67)	22 (10.48)	**.0017**
LEDVT (%)	4 (2.67)	7 (3.33)	.9587
Reintervention (%)	4 (2.67)	19 (9.05)	**.0107**

Data presented as number of subjects (percent of group). The bold value indicates that the comparison between the two groups is statistically significant (*P* < 0.05).

LEDVT = lower extremity deep vein thrombosis, NPWT = negative pressure wound therapy.

### 3.3. Clinical outcome

The long-term follow-up was carried out for the discharged patients. The knee AKSS function scores were obtained at 3 months, 6 months, and 1 year after operation, respectively, to evaluate the knee function recovery of the 2 groups. The results show that there was no significant difference in postoperative function between the 2 groups (Table 3).

**Table 3:** AKSS scores of knee function at follow-up for each group.

AKSS functional score	NPWT group (n = 150)	Non-NPWT group (n = 210)	*P*
3 mo after operation	138.49 ± 31.53	135.66 ± 29.89	.3873
6 mo after operation	144.18 ± 35.24	143.86 ± 34.68	.9317
1 y after operation	148.49 ± 34.65	146.21 ± 36.19	.5490

Data presented as average (standard deviation).

AKSS = American Knee Society Knee Score, NPWT = negative pressure wound therapy.

### 3.4. Cost analysis

**Figure 2.**
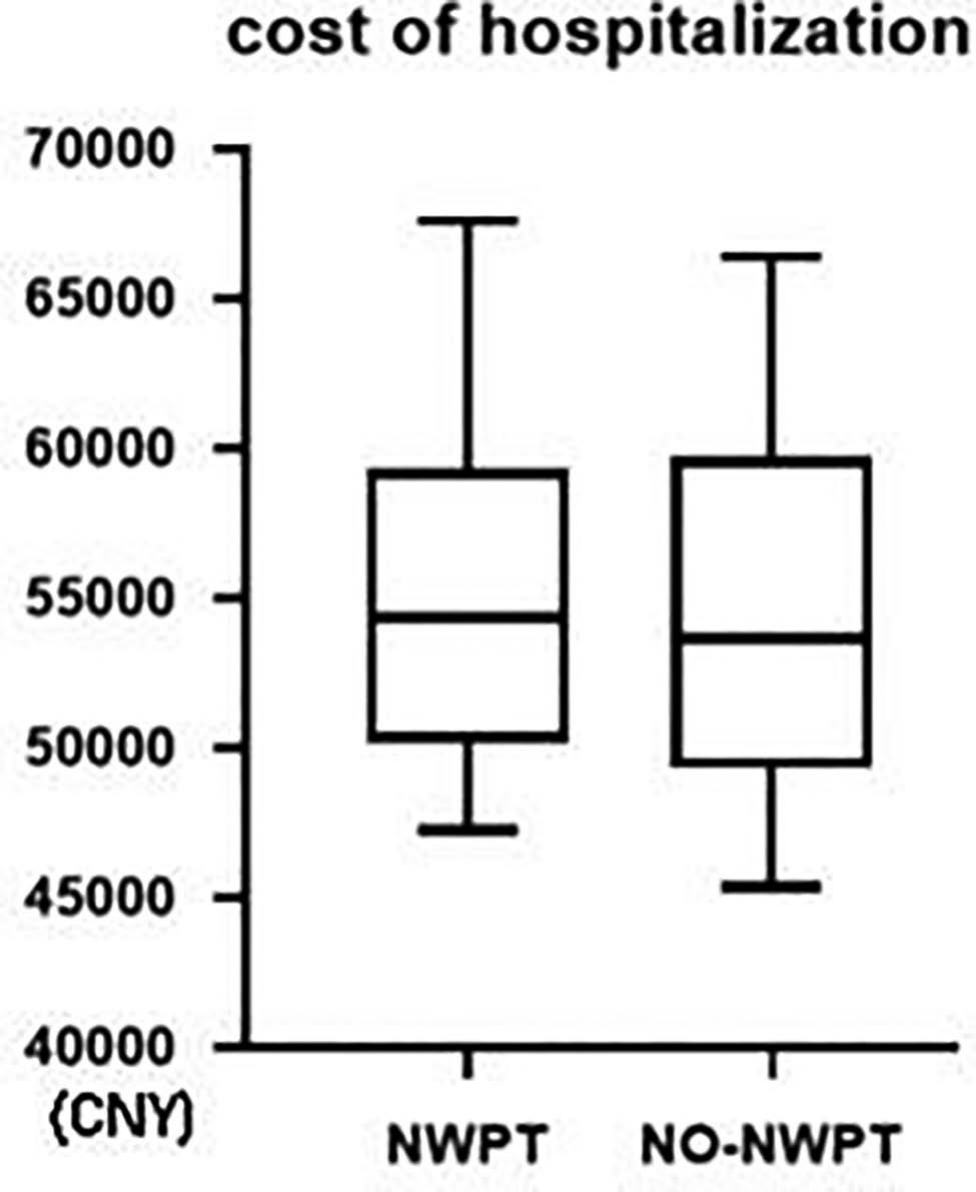
The analysis of cost of hospitalization. There was no significant difference between NPWT group and no-NWPT group in the average total hospitalization cost of knee replacement surgery (55,368 vs 54,946, *P* = .8572). NPWT = negative pressure wound therapy.

No significant difference in the average total hospitalization cost of knee replacement surgery (55,368 vs 54,946; *P* = .8572) was found between the 2 groups, but the maximum and minimum costs of the NPWT group were higher than those of the non-NPWT group (Fig. 2).

## 4. Discussion

Obesity is a well-documented risk factor for the development of osteoarthritis. The performance of joint arthroplasty in individuals with obesity has increased in the last decades. Kerkhoffs et al^[3]^ found that obesity negatively influences the outcome of patients treated with TKA, with more short-term complications and poorer long-term outcome compared with nonobese patients. SSIs and wound complications have been associated with the TKA operations of patients with obesit. Some studies reported increased revision rates, lower functional scores, and increased complication rates, including infections, among patient with obesity following TKA.^[14–16]^ Morbid obesity increases the risk of perioperative complications during TKA, including superficial SSIs and PJI.^[3,15,17]^ Although the exact mechanism is unclear, this phenomenon may be partly explained by a weakened immune response in patients with obesity. The number of monocytes that mature to macrophages is significantly less in patients with obesity.^[18]^ Impaired release of lymphocyte migration inhibiting factor has also been found in insulin-resistant, non-ketotic diabetic, and non-hyperglycemic patients with obesity.^[19]^ Furthermore, obesity has been strongly associated with reduced subcutaneous tissue oxygenation, which is consequently linked to high rates of infection.^[19]^ No complications have been reported in their control group, but 32% of patients with morbid obesity experienced complications. Seventeen percent were superficial SSIs, and 5% were PJI.^[14]^ This result may in part be due to the fact that surgeries in patients with morbid obesity are technically demanding, which prolongs operative time and consequently increases the risk of postoperative infections.^[20,21]^

In the present study, we observed that conventional use of negative pressure devices after TKA reduced the incidence of postoperative superficial SSIs (*P* = .0226), prosthesis infection (*P* = .0331), and dehiscence (*P* = .0227) in patients with obesity. In addition, the probability of returning to surgery were also reduced. However, negative pressure devices also increase the risk of blisters around the wound in patients with obesity. To avoid blisters, we removed the negative pressure suction device 3 days after surgery and adjusted the attraction according to the comfort of the skin around the patient’s knee joint. No significant difference in the incidence of continuous drainage, deep venous thrombosis and AKSS score was found between the 2 groups.

Results of the present study showed that conventional incision NPWT can significantly reduce the incidence of SSIs, wound dehiscence, and reoperation rate in patients with obesity after TKA. The proposed mechanisms by which negative pressure therapy accelerates wound healing include increasing blood flow, enhancing angiogenesis, stimulating growth factors, and removing of interstitial fluid. Closed incisions provide a barrier between the incision and the environment, thus reducing the bacterial burden, and distracting forces thereby maintaining an approximation of wound edges. As a type of wound dressing, it can accelerate wound healing vascular endothelial cells formation. Borgquist et al^[22]^ used a pig model of peripheral trauma to demonstrate that the blood flow of microvessels at the edge of the wound gradually changes with the increase in negative pressure until −80 mm Hg and then reaches a stable state. Increasing blood flow contributes to oxygenation, nutrient supply, and waste clearance, and reducing blood flow stimulates angiogenesis and granulation tissue formation. The best negative pressure level for wound treatment may be the point where the increase in blood flow is balanced with the decrease in blood flow, without causing ischemia or pain. Negative pressure therapy can promote wound healing by controlling microvascular blood flow.^[22]^

Several experiments have been carried out on the treatment of surgical incision and the reduction of infection and other complications with NWPT. Brega et al^[23]^ analyzed 90 patients undergoing cardiac surgery with a median sternal incision and found that, compared with conventional gauze, hydrocolloids, and carboxymethyl fibrin dressings, the patients who underwent NPWT at closed incisions had a significantly lower incidence of deep sternal complications than those who were administered conventional gauze, hydrocolloids, and carboxymethyl fibrin dressings.^[23]^ The difference was obvious in patients with obesity. In a retrospective study, Bonds et al^[24]^ analyzed 254 patients undergoing colorectal surgery and found that 69 (27.2%) developed SSIs, of which 4 (12.5%) occurred in patients receiving incision negative pressure therapy and 65 (29.3%) occurred in patients receiving a standard suture. Multivariate logistic regression analysis showed that negative pressure wound treatment reduced the chance of SSIs (or 0.32; *P* < .05). Obesity has been associated with the increasing trend of SSIs (or 1.64; *P* = .10).^[24]^ However, considering that the cost of vacuum sealing dressing itself is much higher than that of ordinary dressing, NPWT should be used with caution. Yaghmour et al^[25]^ evaluated the use of NPWT in 1403 cases of primary TKA and 279 cases revision TKA. These results indicate that NPWT can significantly reduce the incidence of complications after TKA. However, no significant difference in infection rate was found between the NPWT and control group after primary TKA. The use of NPWT in primary TKA significantly increased the risk of blisters. Therefore, the conventional use of NPWT is not recommended. However, in high-risk patients undergoing revision TKA or primary TKA with high-risk wound problems, NPWT reduces wound complications and may save medical costs.^[25]^ Mueller et al^[26]^ enrolled 274 patients undergoing spinal surgery in a prospective study. The incidence of SSIs with NWPT was significantly lower than that with standard dressings (3.4% vs 10.9%; *P* = .02). No significant difference in the infection rate of simple decompression surgery (4.2% vs 9.1%; *P* = 5.63) was found between the 2 groups, but the infection rate was significantly reduced in the cases requiring internal fixation (3.2% vs 11.4%; *P* = 4.03), but it was significantly reduced in the cases requiring internal fixation (3.2% vs 11.4%; *P* = 5.03).

The incidence of SSIs was lower in high-risk patients using NPWT. Compared with standard dressings, NPWT can significantly reduce the incidence of SSIs in patients undergoing spinal surgery. In view of the severe consequences of infection, the higher cost of NPWT may be reasonable for some high-risk patients undergoing spinal surgery.^[26]^ However, some experiments obtained different results. Nherera et al^[27]^ utilized a randomized controlled trial evaluating the cost-effectiveness of single-use NPWT in patients undergoing primary hip and knee arthroplasty. The cost/patient was $7954 and $9559 for single-use NPWT and standard care respectively resulting in savings of $1607 in favor of single-use NPWT. Greater savings were obtained in sub-groups of high-risk patients with BMI 35 and American Society of Anesthesiologists 3.^[27]^ The cost-benefit evaluation of NPWT produced different results in different indications. Abundant research is ongoing. Decisions about the use of NPWT should take into account surgical indications and the environment, as well as evidence of all outcomes.

Although the present study obtained encouraging results from conventional negative pressure therapy for patients with obesity after TKA, it has certain limitations. First, in addition to hypertension, diabetes and tobacco use, other concomitant medical diseases which may affect the results and cause bias were not considered. Furthermore, these study results are limited by the retrospective nature of the study and hence selection bias, the small sample size, and other confounders which were not accounted for in this study. Finally, we did not have access to general practice data or information as to whether patients returned to different hospitals or were treated with antibiotics for SSIs. Therefore, some wound complications and infections may have been missed, leading to an underestimation of SSIs incidence. Therefore, additional large-scale studies on the use of NPWT in patients with obesity after TKA must be conducted to provide robust evidence for clinical practice.

## 5. Conclusion

This study clearly shows that the use of NPWT can significantly reduce the incidence of complications such as SSIs, wound dehiscence and reoperation rate in patients with obesity after TKA, all of which have beneficial clinical and socio-economic significance.

## Author contributions

Conceptualization: QCS, XQD, LHF.

Data curation: QCS, YZ, GYZ, DLS.

Formal analysis: GYZ, DLS.

Investigation: QCS, DLS.

Methodology: QCS, YZ, GYZ, DLS.

Project administration: QCS, XQD.

Resources: QCS.

Supervision: XQD, QCS.

Validation: DL, XQD.

Writing – original draft: QCS, DL.

Writing – review & editing: QCS, DL, XQD.
